# Supplementary material for: Lower bound for the spatial extent of localized modes in photonic-crystal waveguides with small random imperfections
Source: Sci Rep. 2016 Jun 1;6:27037. doi: 10.1038/srep27037 (PMC4887989; doi:10.1038/srep27037)
Supplement: Supplementary Information [file srep27037-s2.pdf]

Lower bound for the spatial extent of localized modes in photonic-crystal waveguides with small random imperfections: Supplementary Information

Rémi Faggiani[1], Alexandre Baron[2,3,*], Xiaorun Zang[1], Loïc Lalouat[4], Sebastian A. Schulz[5,6], Bryan O'Regan[5,7], Kevin Vynck[1,†], Benoît Cluzel[4], Frédérique de Fornel[4], Thomas F. Krauss[5,7], and Philippe Lalanne[1,2,‡]

[1]LP2N, UMR 5298, CNRS – IOGS - Univ. Bordeaux, 33400 Talence, France
[2]LCF, UMR 8501, CNRS - IOGS - Univ. Paris-Sud, 91127 Palaiseau, France
[3]CRPP, UPR 8641, CNRS - Univ. Bordeaux, 33600 Pessac, France
[4]ICB, UMR 6303, CNRS - Univ. Bourgogne, 21078 Dijon, France
[5]SUPA, School of Physics & Astronomy, University of St Andrews, St Andrews, KY16 9SS, UK
[6]Department of Physics and Max-Planck Centre for Extreme and Quantum Photonics, University of Ottawa, Ottawa, K1N 6N5, Ontario, Canada
[7]Department of Physics, University of York, York, YO10 5DD, UK
[*]alexandre.baron@u-bordeaux.fr
[†]kevin.vynck@institutoptique.fr
[‡]philippe.lalanne@institutoptique.fr

ABSTRACT

This document constitutes supplementary information to support the main manuscript. First, we provide a comprehensive presentation of the numerical method and protocol used to determine the distribution function of the spatial extent of localized mode. The protocol thoroughly eliminates necklace or delocalized states, to solely count states that are truly-confined in a limited space. Second, we present the end-fire transmission characterization of the slow (W1)- and fast (W1.1)-waveguides used to observe a wavelength-scale localized mode as well as a high-resolution scanning electron microscope analysis of the W1 waveguide to seek for abnormally large imperfections that may impact localization in very small areas.

Numerical results

Computational method

A systematic exploration of real PhCWs with long-scale propagation lengths and nanometer perturbations is out of reach of present state-of-the-art 3D computational approaches. For this reason, we resort to a 2D fully-vectorial analysis with an effective index of 2.83 for the guided mode of a 220-nm-thick silicon slab in air. Out-of-plane scattering into the air cladding is therefore omitted in the computation, but since this loss channel is much weaker than the backscattering channel near the band edge, the approximation is likely to impact only weakly our predictions on the spatial extent of the cavity modes.

All our computational results are obtained with an in-house fully-vectorial frequency-domain Fourier-Bloch-mode method.[1,2] The strength of this method is that it relies on an analytical integration of Maxwell's equations along the PhCW axis, allowing us to study long waveguides with an S-matrix formalism which can handle Bloch modes.[2] Another unique feature of the approach is its capability to analytically satisfy the outgoing Bloch-wave conditions at the PhCW termination. This provides a high degree of accuracy to the computational results, even near the band edge frequency. We resort to numerical integration only in the transverse x-direction with a super cell approach and Perfectly-Matched-Layers.[2] The method has already been used for many studies of periodic waveguides and has been compared to experimental investigations of real situations involving PhCWs. For instance, it has been used to study various effects related to the emission of dipole sources in PhCWs[3] and to the transport of light in disordered PhCWs.[4,5]

Individual localized modes

The protocol used to determine whether the resonances observed in the local density of states (LDOS) spectrum correspond to individual localized modes is described in the Methods section. In brief, the field profile of individual localized modes (with spatial extent smaller than the system size) should remain unchanged after extending the waveguide from $100a$ to $200a$ and

their normalized field profile be independent of the source position. Figure S1 shows several examples of resonant modes corresponding or not to individual localized modes. Note that individual localized modes are retrieved independently of the physical mechanism underlying their formation (Anderson localization or gap confinement)[6] and all delocalized modes, including necklace states,[7,8] are excluded.

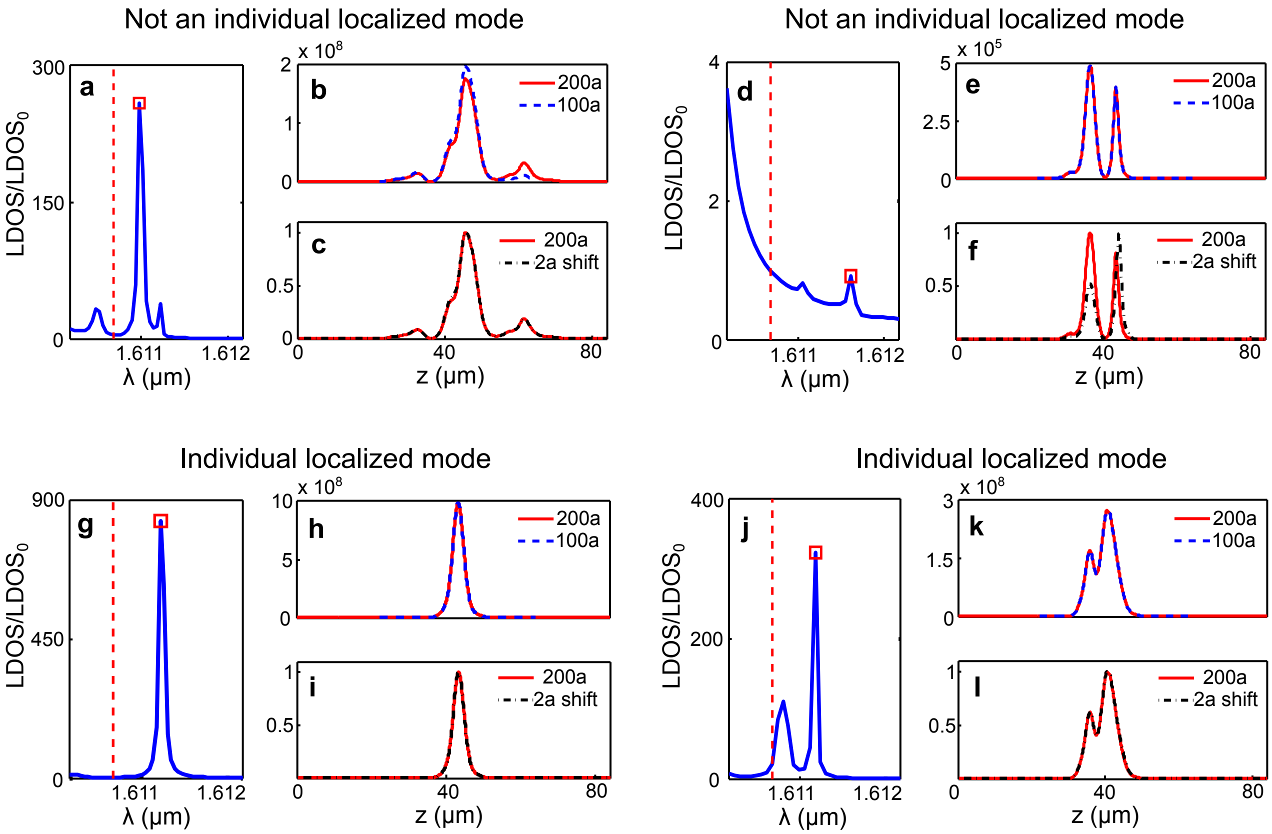

Figure S1. **Examples of resonances that are or are not accounted for in the distribution function of spatial extent of localized modes. (a,d,g,j)** Spectral dependence of the normalized LDOS obtained for a source placed in the center of the $100a$-long W1 waveguide. The vertical red dashed line indicates the band-edge wavelength λ_0. **(b,e,h,k)** Envelop of the magnetic-field-intensity profiles, $|H_{100}|^2$ and $|H_{200}|^2$, at the resonance wavelength for the $100a$-long W1 waveguide (dashed blue line) and the extended $200a$-long W1 waveguide (red solid line). **(c,f,i,l)** Envelop of the magnetic-field-intensity profiles in the $200a$-long W1 waveguide for a source positioned in the center (red solid line) and for the same right-shifted source (black dashed-dotted line). **(a-c)** Resonance that is *not* considered as an individual localized mode (mode profile affected by the boundary). Here, $\sigma = 0.5$ nm. **(d-f)** Resonance that is *not* considered as an individual localized mode (mode profile affected by the source position). Here, $\sigma = 0.75$ nm. **(g-i)** Resonance that is considered as an individual localized mode. The extension length is $L = 6.4$ μm and $\sigma = 0.5$ nm. **(j-l)** Resonance that is considered as an individual localized mode. The extension length is $L = 12.5$ μm and $\sigma = 0.75$ nm.

Characterization of the fabricated sample

Transmission measurements

Prior to near-field characterization, the fabricated sample was characterized via spectroscopic transmission measurements performed at telecommunication wavelengths. As shown in the inset of Fig. S2(a), both the slow (W1) and fast (W1.1) waveguides have access ridge waveguides in order to couple light into the structure. Note also that the first five periods of the waveguides are modified to efficiently couple light into the PhCW mode, independent of the group index. The W1 and W1.1 waveguide transmission spectra were recorded with a high-resolution tunable external laser source ($1450-1620$ nm) using an end-fire setup. The measured spectra are shown as blue and red curves in Fig. S2(a). As expected from the calculated dispersion curves in Fig. S2(b), the W1 transmission drops abruptly near the band-edge wavelength ($\lambda_0 \sim 1490$ nm), while the fast W1.1 waveguide remains transparent for the input signal way beyond λ_0. The bottom black curve corresponds to the transmission

spectrum recorded at the W1 waveguide output when light is injected into the fast W1.1 waveguide. The amount of transmitted light that we observe in the W1 band gap for $\lambda > \lambda_0$ is due to the coupling between the two waveguides. It evidences the possibility of energy transport in the forbidden band of the W1 waveguide over distances that are smaller than the 200-μm waveguide length.

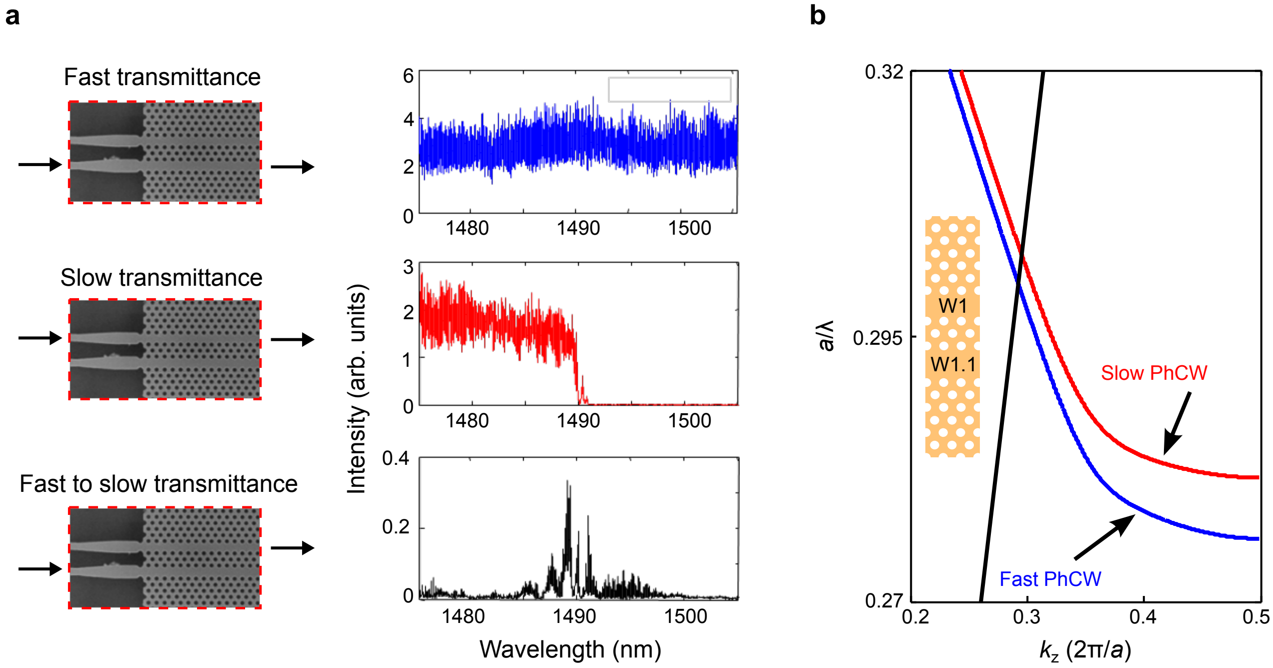

Figure S2. End-fire characterization of the side-coupled PhCWs. (a) Transmission spectra of the W1.1 and W1 waveguides (top blue and middle red curves respectively) when light is injected from the access ridge waveguides. The bottom black curve is the spectrum of the signal collected at the output of the W1 waveguide, when light is injected into the W1.1 waveguide. **(b)** Calculated dispersion curves of the W1 (red) and W1.1 (blue) waveguide for the experimental structural parameters.

High-resolution scanning electron microscope analysis

To assert that the wavelength-scale localized mode reported in the main text is indeed due to the residual imperfections left by our fabrication facility and not to abnormally large imperfections resulting from a failure of the fabrication process, we carefully analyzed the W1 waveguide that produced the localized mode under a high-resolution scanning electron microscope (SEM). Figure S3 shows the three major defects observed in the W1 waveguide which consist in resist stains and small hole deformations. These defects are not observed at the locations of the observed localized mode under the near-field optical microscope shown in the main text. We can thus infer that the formation of localized modes are due to residual imperfections. This conjecture is supported by the computational results that predict that wavelength-scale localized modes exist for disorder levels that are even smaller than those of our fabrication process.

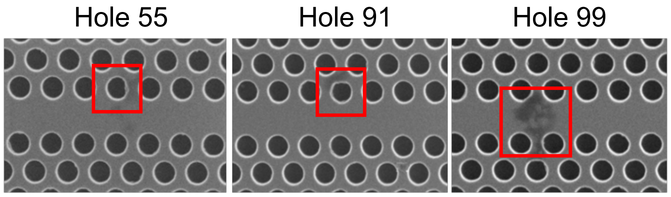

Figure S3. High-resolution scanning electron microscope images of the three major defects in the W1 waveguide exhibiting a localized mode. These imperfections are located at holes number 55, 91 and 99, i.e. at 23.52 μm, 38.22 μm and 41.58 μm respectively from the input facet of the W1 waveguide. Holes 55 and 91 exhibit resist stains and visible impurities around the hole. Hole 99 exhibits a resist stain and severe distortion of the hole.

References

1. Silberstein, E., Lalanne, P., Hugonin, J.-P. & Cao, Q. Use of grating theories in integrated optics. *J. Opt. Soc. Am. A* **18**, 2865 (2001).
2. Lecamp, G., Hugonin, J. P. & Lalanne, P. Theoretical and computational concepts for periodic optical waveguides. *Opt. Express* **15**, 11042 (2007).
3. Lecamp, G., Lalanne, P. & Hugonin, J. P. Very Large Spontaneous-Emission beta Factors in Photonic-Crystal Waveguides. *Phys. Rev. Lett.* **99**, 23902 (2007).
4. Mazoyer, S. *et al.* Statistical fluctuations of transmission in slow light photonic-crystal waveguides. *Opt. Express* **18**, 14654–14663 (2010).
5. Baron, A., Mazoyer, S., Smigaj, W. & Lalanne, P. Attenuation Coefficient of Single-Mode Periodic Waveguides. *Phys. Rev. Lett.* **107**, 153901 (2011).
6. Bliokh, P., *et al.* Colloquium: Unusual resonators: Plasmonics, metamaterials, and random media. *Rev. Mod. Phys.* **80**, 1201–1213 (2008).
7. Bertolotti, J., *et al.* Optical necklace states in Anderson localized 1D systems. *Phys. Rev. Lett.* **94**, 113903 (2005).
8. Sebbah, P., Hu, B., Klosner, J.M. & Genack, A.Z. Extended Quasimodes within Nominally Localized Random Waveguides. *Phys. Rev. Lett.* **96**, 183902 (2006).
